# Short- and Medium-Term Outcomes Comparison of Native- and Valve-in-Valve TAVI Procedures

**DOI:** 10.31083/j.rcm2409255

**Published:** 2023-09-18

**Authors:** Peter V. Bartos, Balazs Molnar, Zoltan Herold, Gabor Dekany, Zsolt Piroth, Gergely Horvath, Abdelkrim Ahres, Christian M Heesch, Nikoletta R. Czobor, Sai Satish, Tunde Pinter, Geza Fontos, Peter Andreka

**Affiliations:** ^1^Department of Cardiology, Gottsegen National Cardiovascular Center, 1096 Budapest, Hungary; ^2^Károly Rácz Doctoral School of Clinical Medicine, Semmelweis University, 1085 Budapest, Hungary; ^3^Division of Oncology, Department of Internal Medicine and Oncology, Semmelweis University, 1083 Budapest, Hungary; ^4^Department of Interventional Cardiology, Florida Heart Clinic, Hallandale Beach, FL 33009, USA; ^5^Department of Interventional Cardiology, Apollo Hospital, 600006 Chennai, India

**Keywords:** aortic stenosis, transcatheter aortic valve implantation, valve-in-valve transcatheter aortic valve implantation, residual mean aortic valve gradient

## Abstract

**Background:**

In high-risk patients with degenerated aortic bioprostheses, valve-in-valve (ViV) transcatheter aortic valve implantation (TAVI) has emerged as a less invasive alternative to surgical valve replacement. To compare outcomes of ViV and native valve (NV) TAVI procedures.

**Methods:**

34 aortic ViV-TAVI performed between 2012 and 2022 using self-expanding valves, were included in this retrospective analysis. Propensity score matching (1:2 ratio, 19 criteria) was used to select a comparison NV-TAVI group from a database of 1206 TAVI procedures. Clinical and echocardiographic endpoints, short- and long-term all-cause mortality (ACM) and cardiovascular mortality (CVM) data were obtained. Subgroup analyses were completed according to the true internal diameter, dividing patients into a small (≤19 mm) valve group (SVG) and a large (>19 mm) valve group (LVG).

**Results:**

Clinical outcomes of ViV- and NV-TAVI were comparable, including device success [88.2% vs. 91.1%, *p* = 0.727], major adverse cardiovascular and cerebrovascular events [5.8% vs. 5.8%, *p* = 1.000], hemodialysis need [5.8% vs. 2.9%, *p* = 0.599], pacemaker need [2.9% vs. 11.7%, *p* = 0.265], major vascular complications [2.9% vs. 1.4%, *p* = 1.000], life-threatening or major bleeding [2.9% vs. 1.4%, *p* = 1.000] and in-hospital mortality [8.8% vs. 5.9%, *p* = 0.556]. There was a significant difference in the immediate post-intervention mean residual aortic valve gradient (MAVG) [14.6 ± 8.5 mm Hg vs. 6.4 ± 4.5 mm Hg, *p <* 0.0001], which persisted at 1 year [*p* = 0.0002]. There were no differences in 12- or 30-month ACM [11.8% vs. 8.8%, *p* = 0.588; 23.5% vs. 27.9%, *p* = 0.948], and CVM [11.8% vs. 7.3%, *p* = 0.441; 23.5% vs. 16.2%, *p* = 0.239]. Lastly, there was no difference in CVM at 1 year and 30 months [11.1% vs. 12.5%, *p* = 0.889; 22.2% vs. 25.0%, *p* = 0.742].

**Conclusions:**

Analyzing a limited group (n = 34) of ViV-TAVI procedures out of 1206 TAVIs done at a single institution, ViV-TAVI appeared to be an acceptable approach in patients not deemed appropriate candidates for redo valve replacement surgery. Clinical outcomes of ViV-TAVI were comparable to TAVI for native valve stenosis.

## 1. Introduction

Transcatheter aortic valve implantation (TAVI) has emerged as an effective treatment for degenerated surgical aortic bioprostheses [1, 2, 3, 4]. In particular, valve-in-valve (ViV) TAVI has been recognized as a safer alternative to re-do surgical aortic valve replacement in patients at high surgical risk [5]. ViV-TAVI currently accounts for approximately 5% of all TAVI procedures performed in the United States [6].

While ViV-TAVI can restore valve function and improve symptoms, the currently limited available data suggest that there may be a higher incidence of certain complications, including transcatheter heart valve (THV) malposition, coronary occlusion, and severe patient prosthesis mismatch (PPM). Further, there may be a higher residual gradient, and post-procedure coronary access is complicated [7, 8, 9, 10, 11, 12, 13].

Given the scarcity of currently published data on ViV-TAVI, the aims of the present investigation were to (1) compare clinical and hemodynamic outcomes of TAVI for aortic stenosis in native valves (NV) with corresponding outcomes in ViV-TAVI procedures; (2) evaluate complication rates for ViV-TAVI and compare the same to NV procedures; and (3) correlate the results with the current literature on the topic to help further define the role of ViV-TAVI in comparison to repeat surgical aortic valve implantation.

## 2. Materials and Methods

### 2.1 Patients

This retrospective single center study included all ViV-TAVI procedures (34 patients) completed between 2012 and 2022 at the National Cardiovascular Institute of Budapest, Hungary. The study project was accepted by the Medical Research Council Scientific and Research Ethics Committee (ETT TUKEB) (IV 1562/2022/EKU), and patients had previously provided written informed consent for the retrospective and anonymized collection of data from the TAVI database. All TAVI implantations had been recommended following a review of the relevant patient data by the Institutional Multi-Disciplinary Heart Team in accordance with institutional best practice guidelines.

A comparison group of native TAVI patients was identified by propensity score matching (PSM). All patients who underwent TAVI for native aortic valve stenosis during the same period were reviewed for potential matching to ViV patients with a 2:1 matching ratio, according to the following criteria: age, body mass index (BMI), sex, baseline New York Heart Association (NYHA) stage, diabetes mellitus, hypertension, atrial fibrillation, previous percutaneous coronary intervention (PCI), previous coronary artery bypass graft (CABG), previous acute myocardial infarction (AMI), porcelain aorta, peripheral artery disease (PAD), coronary artery disease (CAD), previous cardiac pacemaker (PM) or implantable cardioverter-defibrillator (ICD) implantation, previous major stroke and/or transient ischemic attack (TIA), chronic kidney disease (CKD), Society of Thoracic Surgeons Predicted Risk of Mortality (STS-PROM) score, EuroSCORE II., and pre-intervention left ventricular ejection fraction (LVEF), all criteria which previously were shown to have prognostic value [14, 15].

To eliminate potentially confounding factors, patients whose procedure required access other than trans-femoral, and patients with bicuspid valves were excluded from the NV-TAVI cohort.

For ViV interventions, CoreValve Evolut, Evolut Pro and Evolut R (Medtronic) self-expanding (SE) THVs were used. For NV-TAVI procedures, CoreValve Evolut, Evolut Pro, Evolut R (Medtronic) SE-THVs and Acurate Neo (Boston Scientific) SE-THVs were used [16, 17].

### 2.2 Data Collection

**Fig. 1.**
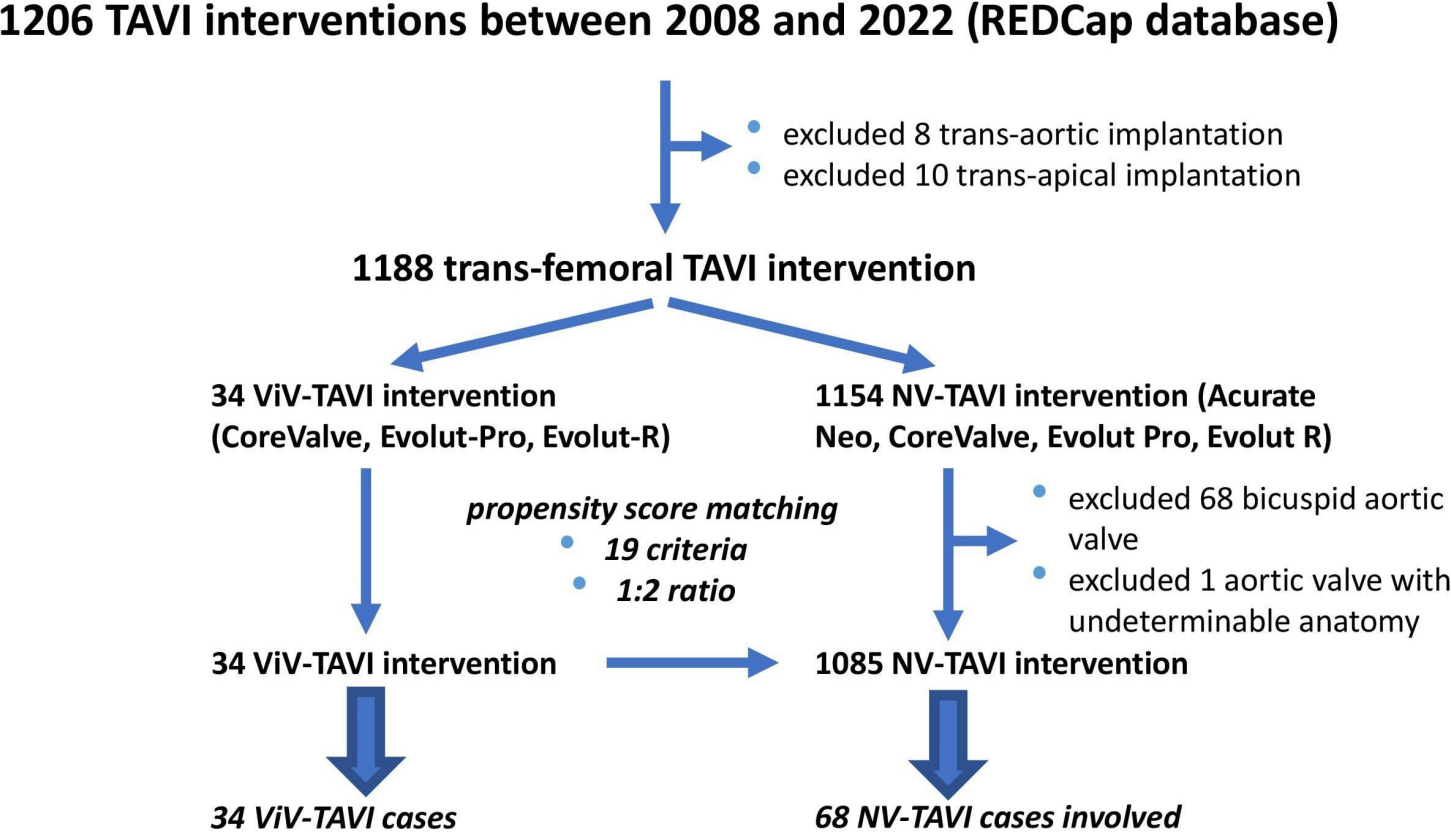
**The data collection process**. TAVI, transcatheter aortic valve implantation; NV, native valve; ViV, valve-in-valve.

Patient data were extracted from the institute’s prospective REDCap database of 1206 TAVI interventions. Groups of 34 ViV-TAVI and 68 NV-TAVI patients were formed (Fig. 1).

Following standard procedures of self-expanding valve implantation, the optimal position of the valve was initially determined by fluoroscopy. A final control was obtained by aortography and transesophageal echocardiography. All procedures were performed through the femoral artery, and procedural details have been described in detail elsewhere [18].

Follow-up data were obtained from hospital medical records and from the patients’ referring cardiologists. After TAVI, transthoracic echocardiography (TTE) was performed prior to discharge, at 6 months, and at 1 year. Data collected included LVEF (%), mean aortic valve gradient (MAVG) (mm Hg), presence and degree of intra- and/or periprosthetic aortic regurgitation (AR) (graded from 1–4), mitral regurgitation (graded from 1–4), and systolic pulmonary artery pressure (sPAP) (mm Hg). Routinely, at 6 and 12 months, the patient functional status was recorded according to NYHA stages. Mortality data were obtained from the Hungarian National Death Registry.

### 2.3 Endpoints

Major clinical endpoints were assessed according to the Valve Academic Research Consortium-2 (VARC-2) criteria [19]. In-hospital mortality reported in the study corresponded to immediate (immediate or sequelae death ≤72 hours after surgery) and subsequent procedural mortality (death beyond 72 hours and within 30 days after surgery or during hospitalization for the index procedure — if postoperative stay longer than 30 days) according to VARC-2. Further, we distinguished between cardiovascular and non-cardiovascular causes of death, as recommended.

Device success was defined by the absence of immediate procedural mortality, correct prosthetic positioning, and intended performance of the THV (no moderate or severe PPM, MAVG <20 mm Hg, peak velocity <3 m/s, and no moderate or severe prosthetic valve regurgitation).

For the purposes of this study, major adverse cardiac and cerebrovascular events (MACCE) (a term which has no universally agreed upon definition) included the most commonly used and potentially fatal components [20]. We reported coronary artery occlusion as defined by VARC-2. Confirmed coronary artery occlusion followed by immediate procedural mortality was considered an acute myocardial infarction.

The severity of AR was rated by TTE and transesophageal echocardiography (TEE) experts according to the VARC-2 criteria, and was not differentiated by origin, i.e., paravalvular vs. transvalvular regurgitation.

We combined hemorrhagic and non-hemorrhagic cerebral infarction in a unified incidence of stroke. Pre-existing CKD was defined by a creatinine level >150 µmol/L. Our reporting of acute kidney injury (AKI) following the procedure included only patients with new hemodialysis requirement.

The STS-PROM score and EuroSCORE II. values were calculated in the standard fashion [21, 22].

Subanalyses were done in ViV patients according to the characteristics of the original aortic bioprosthetic heart valves (BHV). The true internal diameter (ID) of each bioprosthetic valve was determined using the Valve-in-Valve Aortic Application (version 1.0, Minneapolis Heart Institute Foundation, Minneapolis, MIN, USA) [23, 24]. We then grouped the cohort into small bioprosthetic valves (SVG [small valve group] — true ID ≤19 mm) and large bioprosthetic valves (LVG [large valve group] — true ID >19 mm).

### 2.4 Statistical Analysis

The R for Windows software package version 4.2.1 (R Foundation for Statistical Computing, 2022, Vienna, Austria) was used. Matched patient pairs were created using propensity score matching. Comparison of study groups was completed using the Wilcoxon-Mann-Whitney test and the Fisher exact test. *p*-value adjusted pairwise Wilcoxon-Mann-Whitney testing was applied for comparisons between more than two groups. Longitudinal analysis of parameters was performed using linear and logistic mixed effect models. Multinomial logistic regression was used to examine the change over time in the functional status (NYHA) stages. Survival data were analysed using cause-specific competing-risk models [25]. For the multiple comparisons problems, *p*-values were corrected using the Holm method [26]. Continuous and count data were expressed as mean ± standard deviation and the number of observations (percentage), respectively. In addition to the default R procedures, data analyses were performed using lattice (Sarkar, version 0.20-45), lme4 (Bates *et al*. [27], version 1.1-30), Matching (Sekhon & Saarinen, version 4.10-2), nlme (Pinheiro, Bates & R Core Team, version 3.1-159), RcmdrMisc (Fox, version 2.7-2), survival (Therneau, version 3.4-0) and survminer (Kassambara, Kosinski and Biecek, version 0.4.9) R packages [27, 28, 29, 30, 31, 32, 33, 34, 35].

## 3. Results

The majority of the degenerated BHVs (21 patients, 61.76%) were stented-type surgical aortic valves (SAV). The remaining ViV cases consisted of stentless SAV (14.75%), aortic homograft (2.94%), sutureless SAV (14.75%), and THV (5.88%) prostheses. Functionally, 15 patients (44.11%) had stenosis of their bioprosthesis, 7 (20.58%) had significant regurgitation, and 12 (35.29%) presented with both [36, 37, 38] 18 patients (52.95%) had an aortic BHV with a true ID of 19 mm or less. The mean time from surgery to aortic ViV intervention was 8.65 ± 5.35 years.

Among ViV-TAVI patients, significantly more had moderate or severe pre-procedure AR [19 (55.88%) vs. 3 (4.41%), *p* < 0.0001.] ViV and NV-TAVI groups were both at moderate surgical risk (STS-PROM score 5.58% and 4.96%, respectively).

Baseline data on ViV patients (n = 34) and patients with native aortic stenosis (n = 68) after PSM are summarized in Table 1.

**Table 1.** **Baseline characteristics**.

Clinical and echocardiographic variables	ViV-TAVI	NV-TAVI	Crude *p*-value	Adjusted *p*-value
	(n = 34)	(n = 68)		
Age (years)	77.09 ± 8.04	77.05 ± 7.88	0.752	1.000
BMI (kg/$m^{2}$)	29.69 ± 4.88	29.51 ± 5.88	0.717	1.000
Sex (male)	16 (47.06%)	37 (54.41%)	0.532	1.000
EuroSCORE II. (%)	10.96 ± 8.57	9.05 ± 12.04	0.014	1.000
STS-PROM score (%)	5.58 ± 2.72	4.96 ± 3.97	0.075	1.000
NYHA stage average (1–4)	3.35	3.26	0.557	1.000
DM	14 (41.17%)	23 (33.82%)	0.516	1.000
HT	27 (79.41%)	49 (72.05%)	0.478	1.000
HLP	15 (44.12%)	31 (45.58%)	1.000	1.000
AF	7 (20.58%)	17 (25.00%)	0.805	1.000
Previous PCI	10 (29.41%)	21 (30.88%)	1.000	1.000
Previous CABG	13 (38.23%)	32 (47.05%)	0.526	1.000
Previous AMI	9 (26.47%)	20 (29.41%)	0.819	1.000
Porcelain aorta	2 (5.88%)	3 (4.41%)	1.000	1.000
Previous BAV	3 (8.82%)	3 (4.41%)	0.397	1.000
PAD	8 (23.53%)	15 (22.05%)	1.000	1.000
CAD	22 (64.7%)	48 (70.58%)	0.651	1.000
PM/ICD implantation	5 (14.7%)	12 (17.64%)	0.785	1.000
Previous PE	0 (0%)	2 (2.94%)	0.551	1.000
Previous Stroke	3 (8.82%)	9 (13.23%)	0.746	1.000
Previous TIA	2 (5.88%)	0 (0%)	0.108	1.000
COPD	5 (14.70%)	12 (17.64%)	0.785	1.000
CKD	13 (38.23%)	24 (35.29%)	0.828	1.000
LVEF (%)	52.50 ± 13.58	51.41 ± 15.00	0.915	1.000
LVEF ≤30%	4 (11.76%)	8 (11.76%)	1.000	1.000
MAVG (mm Hg)	36.41 ± 16.73	43.75 ± 18.48	0.100	1.000
MAVG ≥20 mm Hg	28 (82.35%)	63 (92.64%)	0.173	1.000
AR moderate/severe (3/4)	19 (55.88%)	3 (4.41%)	*p * < 0.0001	*p * < 0.0001
MR moderate/severe (3/4)	5 (14.70%)	4 (5.88%)	0.266	1.000
TR moderate/severe (3/4)	2 (5.88%)	4 (5.88%)	1.000	1.000

Values are mean ± SD or n (%). ViV, valve-in-valve; TAVI, transcatheter aortic valve implantation; NV, native valve; BMI, body mass index; STS-PROM, Society of Thoracic Surgeons Predicted Risk of Mortality; NYHA, New York Heart Association; DM, diabetes mellitus; HT, hypertension; HLP, hyperlipoproteinaemia; AF, atrial fibrillation; PCI, percutaneous coronary intervention; CABG, coronary artery bypass graft; AMI, acute myocardial infarction; BAV, balloon aortic valvuloplasty; PAD, peripheral arterial disease; CAD, coronary artery disease; PM/ICD, pacemaker/implantable cardioverter defibrillator; PE, pulmonary embolism; TIA, transient ischemic attack; COPD, chronic obstructive pulmonary disease; CKD, chronic kidney disease; LVEF, left ventricular ejection fraction; MAVG, mean aortic valve gradient; AR, aortic regurgitation; MR, mitral regurgitation; TR, tricuspid regurgitation; SD, standard deviation.

In-hospital clinical and hemodynamic outcomes are presented in Table 2.

**Table 2.** **In-hospital outcomes**.

In-hospital clinical outcomes	ViV-TAVI (n = 34)	NV-TAVI (n = 68)	Crude *p*-value	Adjusted *p*-value
In-hospital mortality %	3 (8.82%)	4 (5.88%)	0.556	-
(95% CI)	(2.25%–21.35%)	(1.93%–13.34%)		
Device success (%)	30 (88.2%)	62 (91.1%)	0.727	1.000
MACCE	2 (5.88%)	4 (5.88%)	1.000	1.000
Periprocedural CPR	2 (5.88%)	4 (5.88%)	1.000	1.000
Annulus rupture	0 (0%)	0 (0%)	1.000	1.000
Pericardial tamponade	0 (0%)	0 (0%)	1.000	1.000
Open heart conversion	0 (0%)	0 (0%)	1.000	1.000
Major stroke	0 (0%)	0 (0%)	1.000	1.000
Coronary occlusion	1 (2.94%)	1 (1.47%)	1.000	1.000
Immediate mortality	1 (2.94%)	1 (1.47%)	1.000	1.000
Vascular complications	8 (23.53%)	11 (16.17%)	0.422	1.000
Major vascular complication	1 (2.94%)	1 (1.47%)	1.000	1.000
Bleeding complication	4 (11.76%)	13 (19.11%)	0.410	1.000
Life-threatening or major bleeding	1 (2.94%)	1 (1.47%)	1.000	1.000
AKI (hemodialysis)	2 (5.88%)	2 (2.94%)	0.599	1.000
PP implantation	1 (2.94%)	8 (11.76%)	0.265	1.000
TIA	0 (0%)	0 (0%)	1.000	1.000
Infective endocarditis	1 (2.94%)	2 (2.94%)	1.000	1.000
In-hospital hemodynamic outcomes (TTE)	ViV-TAVI (n = 33)	NV-TAVI (n = 66)	Crude *p*-value	Adjusted *p*-value
LVEF (%)	50.64 ± 11.80	52.77 ± 13.31	0.177	1.000
LVEF ≤30%	3 (9.09%)	6 (9.09%)	1.000	1.000
MAVG (mm Hg)	14.68 ± 8.59	6.43 ± 4.49	*p * < 0.0001	*p * < 0.0001
MAVG ≥20 mm Hg	5 (15.15%)	2 (3.03%)	0.045	0.225
AR moderate or severe (3/4)	0 (0%)	4 (6.06%)	0.298	1.000

Values are mean ± SD or n (%). ViV, valve-in-valve; TAVI, transcatheter aortic valve implantation; NV, native valve; MACCE, major adverse cardiac and cerebrovascular events; CPR, cardiopulmonary pesuscitation; PP, permanent pacemaker; AKI, acute kidney injury; TIA, transient ischemic attack; TTE, transthoracic echocardiography; LVEF, left ventricular ejection fraction; MAVG, mean aortic valve gradient; AR, aortic regurgitation; CI, confidence interval; SD, standard deviation.

Following hospital discharge, five patients (15.62%) in the ViV-TAVI group and 11 patients (16.92%) in the NV-TAVI group were rehospitalized for cardiac causes (*p* = 1.000), most commonly for cardiac decompensation due to permanent atrial fibrillation (AF) (60.0%) in the ViV group and acute coronary syndrome (36.36%) in the non-ViV group. Infective endocarditis incidence [2 patients (6.25%) vs. 3 patients (4.61%), *p* = 1.000] and NYHA stage III or IV at 12 months [6 patients (23.07%) vs. 8 patients (15.09%), *p* = 0.3578] did not differ. 12-month mortality was comparable [ACM (all-cause mortality): 4 patients (11.76%) vs. 6 patients (8.82%), *p* = 0.588; CVM (cardiovascular mortality): 4 patients (11.72%) vs. 5 patients (7.35%),* p* = 0.441].

ViV patients had a significantly higher residual MAVG at 6 and 12 months [6 months: 14.83 ± 8.81 mm Hg vs. 6.96 ± 4.36 mm Hg, *p* < 0.0001; 12 months: 14.00 ± 7.76 mm Hg vs. 6.60 ± 4.19 mm Hg, *p* < 0.0001], whereas the post procedure presence of moderate or severe AR did not differ [6 months: 1 patient (3.44%) vs. 3 patients (5.17%), *p* = 1.000; 12 months: 1 patient (3.84%) vs. 4 patients (7.54%), *p* = 1.000].

Clinical and hemodynamic outcomes of the 12-month follow-up are presented in Table 3.

**Table 3.** **12 months follow-up**.

Survival	ViV-TAVI (n = 34)	NV-TAVI (n = 68)	Crude *p*-value	Adjusted *p*-value
Overall mortality %	4 (11.76%)	6 (8.82%)	0.588	-
(95% CI)	(3.75%–25.0%)	(3.65%–17.0%)		
Cardiovascular mortality %	4 (11.76%)	5 (7.35%)	0.441	-
(95% CI)	(3.75%–25.0%)	(2.75%–15.0%)		
NYHA stage (1–4)	ViV-TAVI (n = 26)	NV-TAVI (n = 53)	Crude *p*-value	Adjusted *p*-value
NYHA average	1.92	1.81	0.552	1.000
Clinical events to 12 months	ViV-TAVI (n = 32)	NV-TAVI (n = 65)	Crude *p*-value	Adjusted *p*-value
Infective endocarditis	2 (6.25%)	3 (4.61%)	1.000	1.000
Cardiac rehospitalisation	5 (15.62%)	11 (16.92%)	1.000	1.000
12 month hemodynamic outcomes (TTE)	ViV-TAVI (n = 26)	NV-TAVI (n = 53)	Crude *p*-value	Adjusted *p*-value
LVEF (%)	53.81 ± 12.61	53.15 ± 14.52	0.933	1.000
LVEF ≤30%	2 (7.69%)	6 (11.32%)	1.000	1.000
MAVG (mm Hg)	14.00 ± 7.76	6.60 ± 4.19	*p <* 0.0001	0.0002
MAVG ≥20 mm Hg	4 (15.38%)	1 (1.88%)	0.039	0.199
AR moderate or severe (grade 3/4)	1 (3.84%)	4 (7.54%)	1.000	1.000

Values are mean ± SD or n (%); ViV, valve-in-valve; TAVI, transcatheter aortic valve implantation; NV, native valve; NYHA, New York Heart Association; TTE, transthoracic echocardiography; LVEF, left ventricular ejection fraction; MAVG, mean aortic valve gradient; AR, aortic regurgitation; CI, confidence interval; SD, standard deviation.

**Fig. 2.**
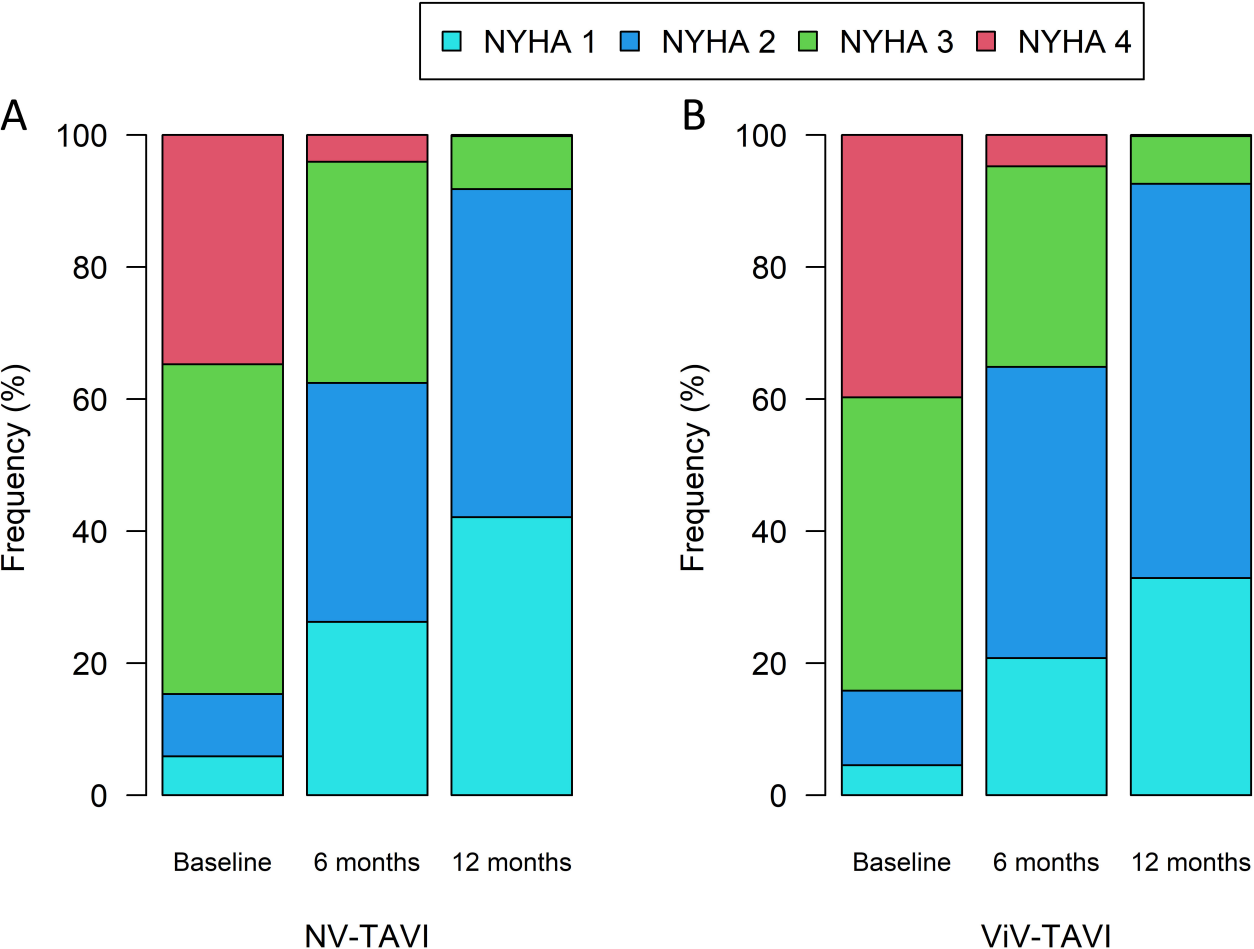
**Changes in NYHA stages of NV-TAVI (A), and ViV-TAVI (B) patients during follow-up**. NYHA, New York Heart Association; NV-TAVI, native valve transcatheter aortic valve implantation; ViV-TAVI, valve-in-valve transcatheter aortic valve implantation.

**Fig. 3.**
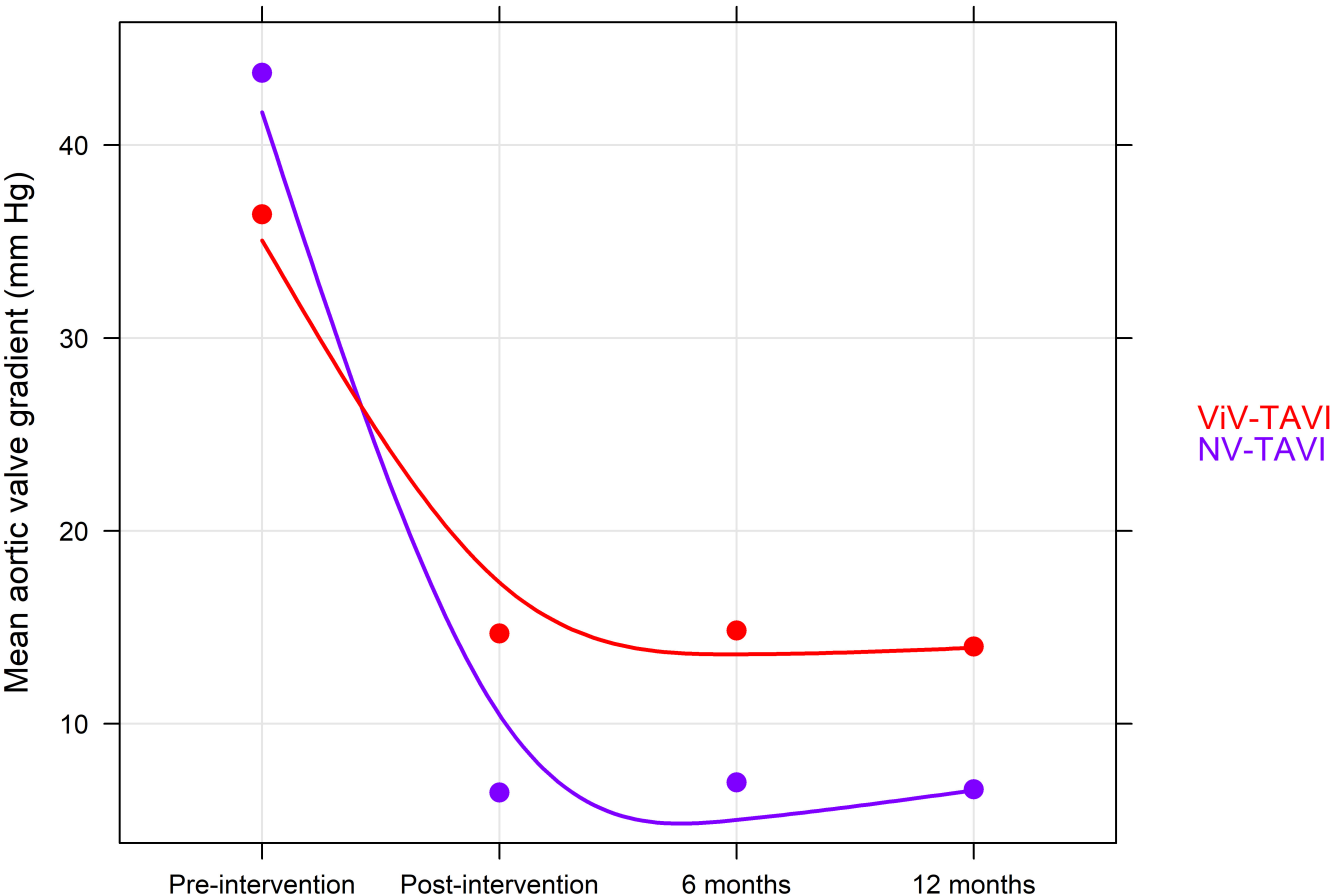
**Change in mean aortic valve gradient during follow-up**. NV-TAVI, native valve transcatheter aortic valve implantation; ViV-TAVI, valve-in-valve transcatheter aortic valve implantation.

Functional status (NYHA stage) improved significantly (*p* < 0.0001) and equally between the two groups (*p* = 0.613) (Fig. 2). MAVG was significantly reduced in both groups post procedure (*p* < 0.0001), in the ViV-TAVI group, this reduction was significantly lower (*p* = 0.036) (Fig. 3).

**Fig. 4.**
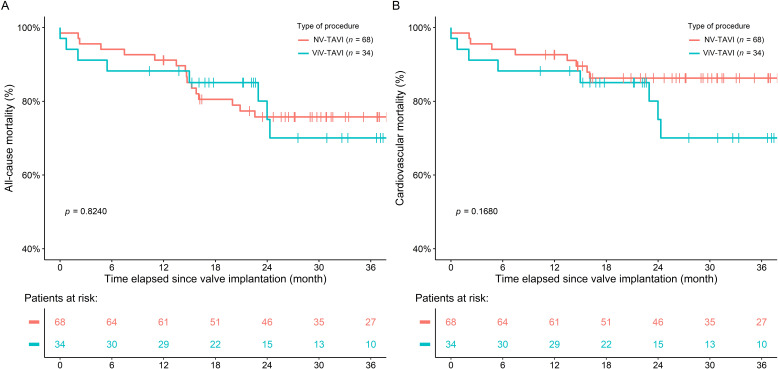
**All-cause (A) and Cardiovascular mortality (B) of patients at 30 months**. NV-TAVI, native valve transcatheter aortic valve implantation; ViV-TAVI, valve-in-valve transcatheter aortic valve implantation.

There was no difference in 30-month survival between the groups [ACM: 8 patients (23.52%) vs. 19 patients (27.94%), *p* = 0.948; CVM: 8 patients (23.52%) vs. 11 patients (16.17%), *p* = 0.239] (Fig. 4). For patients undergoing ViV intervention, only cardiovascular cause of death was recorded.

**Fig. 5.**
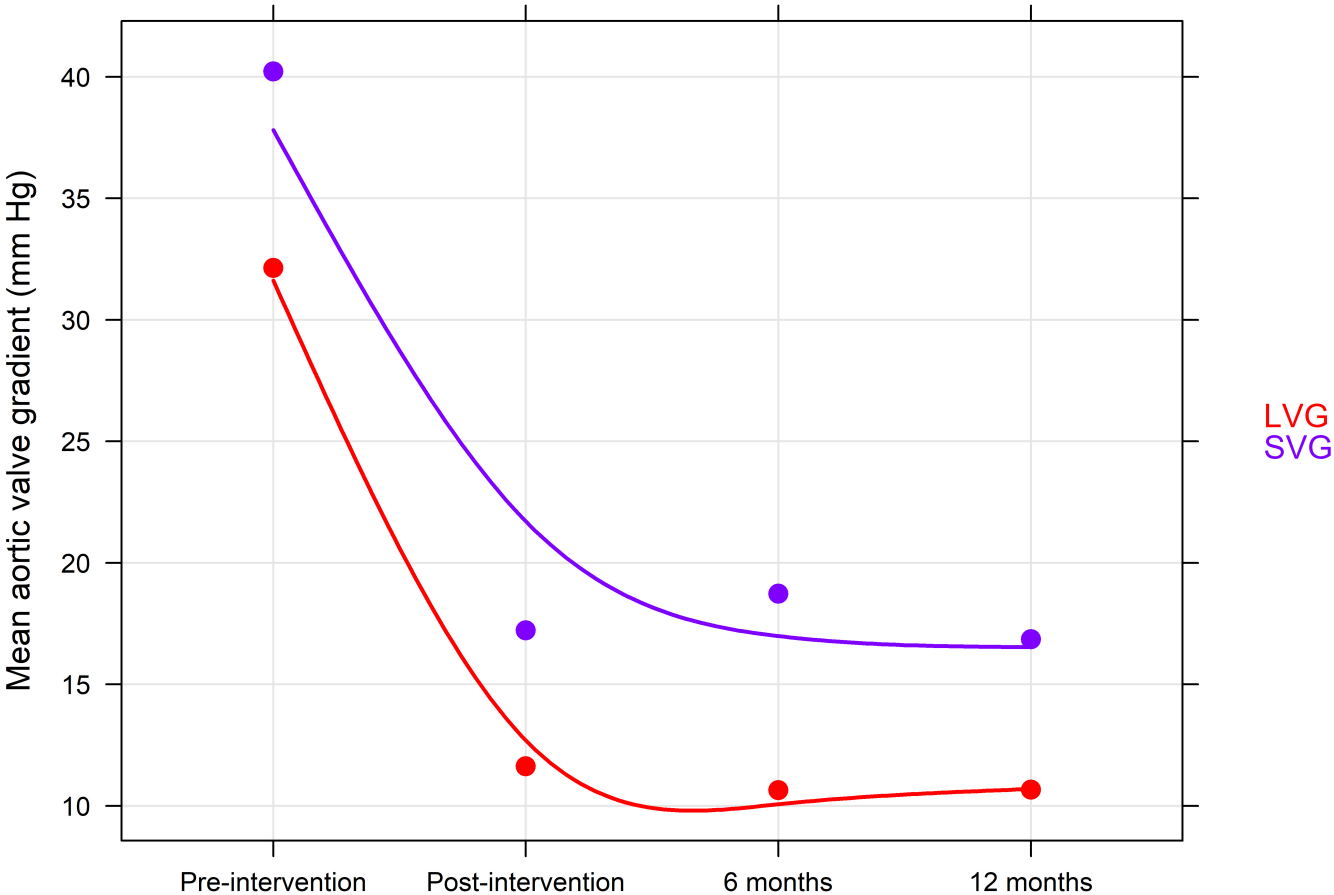
**Change in mean aortic valve gradient according to true internal diameter during follow-up**. LVG, large valve group; SVG, small valve group.

**Fig. 6.**
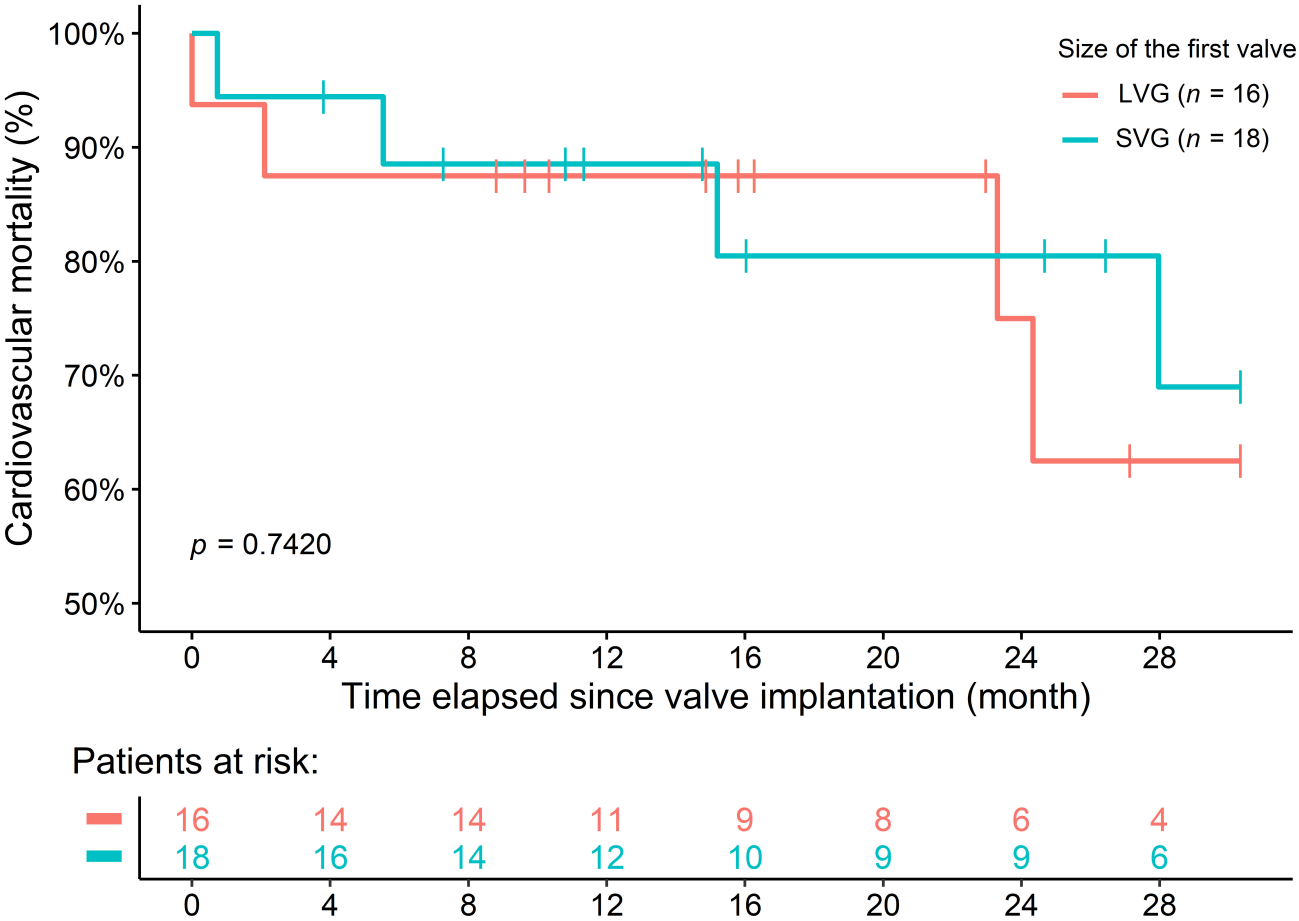
**Cardiovascular mortality of LVG and SVG subgroups at 30-month **. LVG, large valve group; SVG, small valve group.

For the SVG subgroup, we found only numerically higher MAVG compared to LVG before interventions (40.22 ± 11.61 mm Hg vs. 32.13 ± 20.64 mm Hg, *p* = 0.261). However, a significantly higher MAVG was observed for SVG after ViV interventions compared to LVG at 6 months follow up [immediate: 17.2 ± 10. 2 mm Hg vs. 11.6 ± 4.7 mm Hg, *p* = 0.119; 6 months: 18.7 ± 10.1 mm Hg vs. 10.6 ± 4.5 mm Hg, *p* = 0.027; 12 months: 16.8 ± 8.8 mm Hg vs. 10.6 ± 4.7 mm Hg, *p* = 0.0502]. MAVG was significantly reduced in SVG and LVG post procedure (*p* = 0.001), equal between the two subgroups (*p* = 0.948) (Fig. 5). The number of patients with moderate or severe AR also decreased significantly in SVG and LVG (*p* = 0.004), equal between the two subgroups (*p* = 0.252). There was no difference in CVM between the two subgroups based on true ID (SVG and LVG) at 1 year and 30 months [11.11% vs. 12.50%, *p = *0.889; 22.22% vs. 25.0%, *p* = 0.742] (Fig. 6).

## 4. Discussion

Our study groups had comparable risk profiles, allowing the outcome differences between ViV and NV to be attributed with reasonable certainty to the ViV vs. NV aspect of the procedure. We did find that the mean residual aortic pressure gradient was significantly higher at 6 months and 12 months in patients undergoing ViV implantation at 6 months and 12 months, but the degree of aortic insufficiency noted was comparable.

While our total number of ViV-TAVI procedures in this single center study was, of course, limited,ViV-TAVI was a feasible and safe strategy for the treatment of degenerated BHVs in terms of in-hospital, 6-month and 12-month clinical outcomes. These results comport with the findings of a recently published meta-analysis involving 1442 patients undergoing ViV-TAVI and 6986 patients undergoing NV-TAVI interventions [7].

In-hospital and 1-year mortality rates following ViV-TAVI were 8.8% and 11.8%, comparable to those observed in the NV-TAVI group. In our patients, there were no major strokes or TIAs observed immediately or during the hospital stay, and our device success rate was comparable between ViV and NV-TAVI groups (88.23% vs. 91.17%), findings also in agreement with the literature [1, 38, 39, 40, 41, 42, 43, 44, 45].

A direct comparison of survival rates between our ViV-TAVI patient group and reported survival rates of patients undergoing redo-surgical valve replacement is difficult, since TAVI patients in general, and ViV-TAVI patients in particular, are selected for TAVI since they have a lower life expectancy and higher operative risk then surgical candidates. A recent series of contemporary outcomes in repeat aortic valve surgery patients showed short term mortality data similar to our ViV-TAVI group (9.5%), but long-term outcomes far superior (74% survival at 5 years in the published surgical patient series) [46]. This is in accordance with a recent review and meta analysis of ViV-TAVI versus surgical redo aortic valve replacement publications, showing a reduction in mortality of 30% for ViV-TAVI, but, inter alia, less severe patient-prosthesis mismatch in the surgical group, and higher post-operative aortic valve gradients in the ViV-TAVI group [47].

As expected, our patient groups were older (77 years on average for both groups). Further, BMI was 29.69 for the ViV group and 29.51 for the NV group. Malnutrition and frailty have recently emerged as important factors (possibly amenable to corrective intervention) for long-term outcomes after TAVI [48, 49]. While the degree of patients’ frailty was not comprehensively assessed in our patient database, judging by their similar age and BMI values, a significant difference in this important marker was not likely present between groups.

Our subgroup analysis, as expected, shows that the presence of small BHVs (true ID ≤19 mm) is associated with significantly higher gradients after ViV intervention, a factor to be considered when selecting the appropriate treatment mode for patients with degenerated small surgically placed aortic valves [50, 51, 52, 53, 54]. In fact, the higher MAVG that was (predictably) noted post-procedure in our ViV group reemphasizes the need to consider annulus enlargement techniques in small native annulus patients at the time of primary surgical valve placement, and to strongly consider redo-surgery in patients presenting with degenerated surgically placed aortic valve prostheses [55].

Bioprosthetic valve fracture techniques have recently emerged as a possible solution to the small annulus size of surgically implanted valves [56]. In our series of 34 ViV-TAVI procedures, only two valves were fracturable. Further, our patient series begins in 2012, whereas the procedure gained popularity only at the end of the last decade. Looking forward, however, fracturing of suitable valves now is accepted as an adjunct procedure to ViV that may have a key role in improving hemodynamics. Lastly, following NV-TAVI, coronary occlusion is a rare (<1%) but life-threatening complication [57, 58]. While initial data had suggested that this might be up to four times more common (~3.5%) during ViV-TAVI [59], such a difference in the cohorts was not noted, in keeping with newer data reported by others [2, 3, 7, 60].

Our study has several limitations. Despite the application of propensity score matching and the use of appropriate exclusion criteria, the possible influence of other potentially confounding factors cannot be excluded. Furthermore, although the data analysis included all patients undergoing ViV-TAVI in this single-center study, the number of patients undergoing ViV implantation was relatively low.

## 5. Conclusions

In summary, our data, obtained over a span of 15 years from a single, large cardiovascular center, suggest that ViV-TAVI is a reasonable treatment option with a risk profile comparable to NV-TAVI procedures. A higher MAVG, especially for SVG, was noted, but it was not associated with a short- or medium- term survival disadvantage.

## Data Availability

The anonymized data that support the findings of this study are available from the first author (PVB) upon request following institutional patient privacy guidelines.

## Abbreviations

ACM, all-cause mortality; AF, atrial fibrillation; AKI, acute kidney injury; AMI, acute myocardial infarction; BAV, balloon aortic valvuloplasty; BHV, bioprosthetic heart valve; BMI, body mass index; BVF, bioprosthetic valve failure; CABG, coronary artery bypass grafting; CAD, coronary artery disease; CKD, chronic kidney disease; COPD, chronic obstructive pulmonary disease; CPR, cardiopulmonary resuscitation; CVM, cardiovascular mortality; DM, diabetes mellitus; HLP, hyperlipoproteinaemia; HT, hypertension; ICD, implantable cardioverter defibrillator; ID, internal diameter; LVEF, left ventricular ejection fraction; LVG, large valve group; MACCE, major adverse cardiac and cerebrovascular events; MAVG, mean aortic valve gradient; MR, mitral regurgitation; NV, native valve; NYHA, new york heart association; PAD, peripheral arterial disease; PCI, percutaneous coronary intervention; PE, pulmonary embolism; PP, permanent pacemaker; PPM, patient prosthesis mismatch; PSM, propensity score matching; SAV, surgical aortic valve; SD, standard deviation; SE, self-expanding; sPAP, systolic pulmonary artery pressure; STS-PROM, society of thoracic surgeons predicted risk of mortality; SVG, small valve group; TAVI, transcatheter aortic valve implantation; THV, transcatheter heart valve; TIA, transient ischemic attack; TR, tricuspid regurgitation; TTE, transthoracic echocardiography; ViV, valve-in-valve.
